# Biosorption of uranium by immobilized *Nosto*c sp. and *Scenedesmu*s sp.: kinetic and equilibrium modeling

**DOI:** 10.1007/s11356-022-21641-9

**Published:** 2022-06-30

**Authors:** Mostafa M. S. Ismaiel, Yassin M. El-Ayouty, Saad A. Abdelaal, Hoda A. Fathey

**Affiliations:** 1 Department of Botany and Microbiology, Faculty of Science, Zagazig University, Zagazig, 44519 Egypt; 2 Nuclear Research Center, Egyptian Atomic Energy Authority, P.O. Box, 13759, Cairo, Egypt

**Keywords:** Chlorophyta, Cyanophyta, Biosorption, Uranium, Isotherms, Kinetics

## Abstract

Different activities related to uranium mining and nuclear industry may have a negative impact on the environment. Bioremediation of nuclear pollutants using microorganisms is an effective, safe, and economic method. The present study compared the uranium biosorption efficiency of two immobilized algae: *Nostoc* sp. (cyanophyte) and *Scenedesmus* sp. (chlorophyte). Effects of metal concentration, contact time, pH, and biosorbent dosage were also studied. The maximum biosorption capacity (60%) by *Nostoc* sp. was obtained at 300 mg/l uranium solution, 60 min, pH 4.5, and 4.2 g/l algal dosage, whereas *Scenedesmus* sp. maximally absorbed uranium (65 %) at 150 mg/l uranium solution, 40 min, pH 4.5, and 5.6 g/l of algal dosage. The interaction of metal ions as Na_2_SO_4_, FeCl_3_, CuCl_2_, NiCl_2_, CoCl_2_, CdCl_2_, and AlCl_3_ did not support the uranium biosorption by algae. The obtained data was adapted to the linearized form of the Langmuir isotherm model. The experimental *q*_max_ values were 130 and 75 mg/g for *Nostoc* sp. and *Scenedesmus* sp., respectively. Moreover, the pseudo-second-order kinetic model was more applicable, as the calculated parameters were close to the experimental data. The biosorbents were also characterized by Fourier-transform infrared spectroscopy (ATR-FTIR), energy-dispersive X-ray spectroscopy (EDX), and scanning electron microscopy (SEM) analyses. The results suggest the applicability of algae, in their immobilized form, for recovery and biosorption of uranium from aqueous solution.

**Supplementary Information:**

The online version contains supplementary material available at 10.1007/s11356-022-21641-9.

## Introduction

Uranium is a radioactive element that can be found in different environmental sources including water, soil, and air (Gok and Aytas 2009; Monti et al. 2019; Yue et al. 2021; Gandhi et al. 2022; Smječanin et al. 2022). The importance of this element in many industries like electricity production and medical applications increased the mining and milling processes to acquire it in a considerable amount (Awan and Khan 2015; Yue et al. 2021). However, these processes may lead to uranium leakage and therefore increase its limit above the allowed dose in nature causing serious environmental issues in addition to health hazards for living organisms (Monti et al. 2019; Yue et al. 2021). The hazards may include harmful effects on the nervous system, spleen, kidney, nephrons, liver, and lungs and ultimately cause cell malfunction or cancer. Moreover, exposure to uranium causes allergic reactions, dermatitis, and weakness of the immune system of living organisms where it binds with proteins and anions forming complex inside the body (Schnug and Haneklaus 2008; Monti et al. 2019). In addition, the high binding affinity between DNA molecules and uranium resulted in genotoxic effects (Farooq et al. 2010). This emphasizes the importance of uranium remediation in a safe mode from the environment.

Chemical precipitation, ion exchange, evaporation concentration, membrane separation, adsorption, and other traditional physical and chemical procedures are among the most regularly utilized processes to clean up uranium-contaminated wastewater (Gok and Aytas 2009; Yue et al. 2021). Nonetheless, the physical approach was the sole applicable choice for uranium remediation, from contaminated water. This may be due to financial and technical limitations, in addition to the dangerous by-products resulting from the other methods (Yue et al. 2021).

The biosorption process can be defined as the capability of biological materials to uptake metal ions from wastes through the chemical and physical removal of metal ions. Remediation of heavy metals and toxic pollutants using biological materials like algal biomass is a reliable, flexible, cheap, and friendship method compared with the conventional ways (Gavrilescu et al. 2009; Smječanin et al. 2022). The efficiency of metal removal using algal biomass is affected by several factors like algal species, metal ion charges, and components of the heavy metal solution. In addition, the pH, biomass dose, temperature, and concentration of metal ions have a great effect on biosorption rate (El-Naas et al. 2007; Bayramoglu et al. 2015; Ahmad et al. 2018).

Another factor is the interference of metals in the natural wastes for the biosorption process. This raises the need for more investigations for the optimization conditions to efficiently uptake the metal of interest, like uranium, from waste streams (El-Naas et al. 2007; Gok and Aytas 2009). Amini et al. (2013) reported that the presence of metal ions, beside that of interest, in the solution may interfere with the removal efficiency due to competition on active sites and so decreasing or preventing the removal of metal of interest. They observed negligible effect of most tested cations and anions on the removal of uranium by *Chlorella vulgaris* except Al^+3^ which decreased the uranium removal. It was reported that Cu^2+^, Ni^2+^, Zn^2+^, Cd^2+^, and Mn^2+^ competed slightly with uranyl ions for removal efficiency using *Scenedesmus obliquus* (Zhang et al. 1997).

Several studies have used free microalgae as an absorbent for uranium, including cyanobacteria as *Spirulina platensis* and *Nostoc linckia* (Cecal et al. 2012), *Synechococcus elongatus* (Acharya et al. 2009), *Anabaena flos-aquae* (Yuan et al. 2020), in addition to the chlorophytes (green algae) *Scenedesmus obliquus* (Zhang et al. 1997), *Chlorella salina* (Manikandan et al. 2011), *Chlorella vulgaris* (Amini et al. 2013), *Chlamydomonas reinhardtii* (Erkaya et al. 2014), *Botryococcus braunii* (Celik et al. 2019), *Parachlorella* sp. AA1 (Yoon et al. 2021), and the haptophyte *Isochrysis galbana* (Manikandan et al. 2011). However, different factors may restrain the algal biosorption efficacy including small size, low density, low mechanical strength, and ease of handling (Kadimpati 2017). Immobilization enables microalgae to be used efficiently in different ways including the removal of organic pollutants, heavy metals, and nutrients from wastes, extraction of metabolites from culture media, simple biomass collection, simple regeneration, ease of solid-liquid separation, and friendly re-usable facility (Kadimpati 2017; Ahmad et al. 2018; Mallick 2020).

Tobilko et al. (2008) reported the high effectiveness of *Scenedesmus acutus*, *Chlorella vulgaris*, *Microcoleus vaginatus*, and *Neocystis broаdiensis* biomass for uranium sorption when mixed with clay minerals (montmorillonite) at pH 6 for 1h. Also, Erkaya et al. (2014) investigated free and carboxymethyl cellulose (CMC)-entrapped *C. reinhardtii* cells. Yet, the biosorption efficiency of free algal cells (337.2 mg U(VI)/g) surpassed both CMC-entrapped cells (196.8 mg U(VI)/g) and bare CMC beads (153.4 mg U(VI)/g). Bayramoglu et al. (2015) introduced the polyethyleneimine- (PEI) and amidoxime-modified *Spirulina platensis* biomasses for the removal of uranium ions in batch conditions. They reported the rapid removal of ions by the modified algal biomass compared to the native one. Moreover, Liu et al. (2022) reported a new chitosan/*Chlorella pyrenoidosa* composite adsorbent bearing phosphate ligand. This composite has high uranium adsorption efficiency at a pH of 5. However, the traditional alginate method still be regarded as a simple and efficient detoxificant matrix (Gok and Aytas 2009; Kadimpati 2017). Yet, further investigations were required regarding the biosorption efficacy of uranium by alginate-immobilized microalgae.

In this work, the biosorption of uranium was investigated by two different immobilized microalgae (a cyanophyte and a chlorophyte) to evaluate and compare their biosorption efficiency. In addition, the effects of different uranium concentrations, contact time, pH, biomass dose, and interference of other metal ions on uranium biosorption were also discussed. Furthermore, the experimental data were analyzed by adsorption isotherms, equilibrium, and kinetics models to understand the physicochemical aspects of biosorption and to evaluate their application on large scale. Finally, the surface characterization of immobilized algal biomass, before and after the biosorption process, was examined as well.

## Materials and methods

### Algal strains and culture media

Two microalgae were investigated in this work, the cyanophyte *Nostoc* sp. and the chlorophyte *Scenedesmus* sp., obtained from Voucher specimen of Phycology Lab., Department of Botany and Microbiology, Faculty of Science, Zagazig University (Fig. S1). The modified Watanabe medium (Watanabe 1951; as modified by El-Nawawy et al. 1958) and BG11 (Stanier et al. 1971) were used for growing and maintaining *Nostoc* sp. and *Scenedesmus* sp., respectively (sup. Table S1). The pH was adjusted to 7.4 and 7.1 for modified Watanabe and BG11 media, respectively, with 1N of NaOH or HCl. After which, 245 ml of the standard media was poured into 500-ml-size Erlenmeyer conical flasks, autoclaved at 121°C for 20 min, cooled, and inoculated (under aseptic condition) with 5 ml of previous algal cultures (from the mid-log phase). After that, the cultures were incubated at 27°C ± 2 with a continuous light intensity of 95 μmol photons m^−2^ s^−1^ for 10 days. The cultures were gently shaken twice daily by hand.

### Preparation of immobilized algal cells

To entrap the algal cells into the alginate matrix, the procedure of Chen (2001) was followed. Firstly, the solution of sodium alginate (Lanxess Co., Cologne, Germany) was prepared (4 g/100 ml hot dH_2_O) and autoclaved at 121°C for 20 min. The algal cells (in log phase) were harvested by centrifugation of the grown algal culture at 5000 *x*g for 10 min and washed twice with sterile dH_2_O. The harvested algal cells were thoroughly mixed with the sodium alginate (4%) solution (at ambient temperature) to obtain a cell suspension of ≈ 2 × 10^7^ cells ml^−1^. The algal beads (≈4 mm in diameter) were formed by dropping the algal-alginate solution into 0.03 M CaCl_2_ solution at ambient temperature using a burette (≈8 beads were formed/1 ml algal-alginate solution). The formed beads were left to harden for 30 min, washed with sterile dH_2_O to get rid of excess CaCl_2_, and immediately sealed and stored solely in absolute darkness (by wrapping the container with paper foil) at 4°C until used. A constant algal fresh weight (FW) (1.4 g/l; entrapped inside the beads) was used for the next experiments (Fig. S2).

### Preparation of uranium solution

The stock uranium (uranyl nitrate UO_2_(NO_3_)_2_; Columbus Chemical Ind., Arizona, USA) solution was prepared by the laboratories of the Nuclear Materials Authority, Cairo, Egypt, by dissolving 0.5 g of the uranyl nitrate in 250 ml of de-ionized water. The concentration of uranium in this stock was measured and then diluted to give the final concentrations used in this study.

### Determination of uranium

The uranium concentration was analyzed via the modified method of Sakharov (1974) as described by Davies and Gray (1964). In brief, the samples (5 ml) were put in 100-ml-size Erlenmeyer flasks, and 10 ml phosphoric acid (H_3_PO_4_, 85%) was added and shaken to mix, followed by 1ml of concentrated HCl and 5 drops of 10% ammonium ferrous sulfate. After that, 3 drops of 15% TiCl_3_ were added which turned the solution to violet color. The mixture was left for 5 min and another 3 drops of 15% NaNO_2_ and 5ml urea (20%) were added followed by rapid shaking till the disappearance of effervescence. The mixture was left for 2 min and then 2 drops of the diphenylamine sulfonate indicator (0.2 g diphenylamine 4-sulfonic acid sodium salt mixed with 0.2 g sodium carbonate and dissolved by stirring in dH_2_O to a final volume of 100 ml) were added. The samples were finally titrated against ammonium vanadate (0.001 M NH_4_VO_3_) till the appearance of pale violet color.

The uranium concentration was calculated via the following equation

$$\mathrm{U}\left(\mathrm{mg}/\mathrm{l}\right)=\left(\mathrm{T}\ast {\mathrm{V}}_1\ast 1000\right)/\mathrm{V}$$

where *T* is the molarity of NH_4_VO_3_ solution (i.e., 0.001 M), *V*_1_ is the consumed volume of NH_4_VO_3_, and *V* is the volume of the measured sample.

### Factors affecting the uranium biosorption process

#### Effect of initial uranium concentration

Different concentrations of uranium (50, 100, 125, 150, 200, 300, and 400 mg/l) were prepared, as above, to follow their effect on uranium biosorption by the immobilized algae based on preliminary experiment. Twenty-five milliliters of each concentration was mixed with algal beads (contained 0.035g FW; equivalent to1.4 g/l) in 125-ml-size Erlenmeyer flasks. Triplicate sets were prepared and the flasks were shaken at 100 rpm for 2 h at 27°C. The algal beads were filtrated using a liquidator, and the filtrate was centrifuged at 5000 *x*g and kept for the measurement of residual uranium concentration.

#### Effect of contact time

In this experiment, 25 ml of uranium solutions (150 and 300 mg/l for *Scenedesmus* sp. and *Nostoc* sp. respectively) was mixed with algal beads (0.035g FW). Triplicate sets were prepared and the flasks were shaken at 100 rpm for different times (5, 10, 15, 20, 25, 30, 40, 50, 60, and 75 min) at 27°C. The filtrate was prepared for uranium determination as discussed above.

#### Effect of pH on uranium biosorption

Twenty-five milliliters of uranium solutions (150 and 300 mg/l for *Scenedesmus* sp. and *Nostoc* sp., respectively) was added in 125-ml-size Erlenmeyer flasks. The pH of the solutions was adjusted to different values (3.5, 4.5, 5.5, 6.5, 7.5, and 8.5) with 1N of NaOH or HCl; and then mixed with algal beads (0.035 g FW). Triplicate sets were prepared and the flasks were shaken at 100 rpm for 40 and 60 min for *Scenedesmus* sp. and *Nostoc* sp., respectively, at 27°C. The filtrate was cleared for uranium determination as discussed above.

#### Effect of different biomass dose

Twenty-five milliliters of uranium solutions (150 and 300 mg/l for *Scenedesmus* sp. and *Nostoc* sp. respectively) was added in 125-ml-size Erlenmeyer flasks. The pH of uranium solutions was adjusted to 4.5 and then mixed with algal beads of different fresh algal weights (0.035, 0.07, 0.105, 0.14, and 0.175 g/25 ml, which is equivalent to 1.4, 2.8, 4.2, 5.6, and 7 g/l). Triplicate sets were prepared and the flasks were shaken at 100 rpm for 40 and 60 min for *Scenedesmus* sp. and *Nostoc* sp., respectively, at 27°C. The residual uranium was then examined.

### Optimization of conditions for uranium biosorption efficiency

The best conditions obtained from the above investigated factors, for uranium biosorption by algae, were combined in this experiment. In brief, 25 ml of uranium solutions was prepared (150 and 300 mg/l for *Scenedesmus* sp. and *Nostoc* sp. respectively); the pH was adjusted to 4.5, and then mixed with algal beads (0.14 and 0.105 g FW; equivalent to 5.6 and 4.2 g/l) of *Scenedesmus* sp. and *Nostoc* sp., respectively). Triplicate sets were prepared and the flasks were shaken at 100 rpm for 40 and 60 min, for *Scenedesmus* sp. and *Nostoc* sp. respectively, at 27°C. Finally, the uranium concentration was determined.

### Interference of metal ions with uranium biosorption

The experiment was conducted under the optimum conditions of uranium biosorption (as mentioned above) to study the influence of different concentrations of Na_2_SO_4_ (5680, 11360, 22720, 45440, and 71000 mg/l), FeCl_3_, CuCl_3_, NiCl_3_, CdCl_3_ (10, 20, 30, and 50 mg/l), and AlCl_3_ (53, 107, 160, 213, and 277 mg/l) on biosorption efficiency of uranium by immobilized *Scenedesmus* sp. and *Nostoc* sp. as compared with the control (no added metal). The flasks include 25 ml of uranium concentration of 150 and 300 mg/l (for *Scenedesmus* sp. and *Nostoc* sp. respectively), pH adjusted to 4.5, and algal beads (5.6 and 4.2 g FW/l of *Scenedesmus* sp. and *Nostoc* sp. respectively). Triplicate sets were prepared and the flasks were shaken at 100 rpm for 40 and 60 min for *Scenedesmus* sp. and *Nostoc* sp. respectively at 27°C. Next, the residual concentration of uranium was quantified.

### Calculation of adsorbed uranium

The amount of adsorbed uranium ions per unit of adsorbent (algal beads) was obtained by using the following equation:

1 $$q_e=\left(C_i-C_e\right)\times V/M$$

where *q*_e_ is the amount of uranium adsorbed onto the unit mass of the beads (adsorbent) (mg/g), *C*_i_ and *C*_e_ are the concentrations of the uranium ions before and after biosorption (mg/l), *V* is the volume of the uranium solution (l), and *M* is the amount of the adsorbent (g).

The percentage of uranium removal was calculated as follows:

2 $$\mathrm{Uranium}\;\mathrm{removal}\%=\left(C_{\mathit i}-C_{\mathit e}\right)/C_{\mathit i}\times100$$

### The Langmuir adsorption isotherm

The Langmuir adsorption isotherm describes the surface of the adsorbent as a homogeneous layer, assuming that there is no lateral interaction between the adjacent adsorbed molecules, as a single molecule occupies a single site on the adsorbent surface (Liu et al. 2019)

3 $${q}_e={q}_{\mathrm{max}}{K}_{\mathrm{L}}{C}_{\mathrm{e}}/1+{K}_{\mathrm{L}}{C}_{\mathrm{e}}$$

The Langmuir’s isotherm (Eq. 3) was linearized to determine the adsorption parameters as follows:

4 $$1/{q}_e=\left(1/{K}_{\mathrm{L}}{q}_{\mathrm{max}}\right).\left(1/{C}_{\mathrm{e}}\right)+\left(1/{q}_{\mathrm{max}}\right)$$

where *q*_max_ is the maximum adsorption capacity (mg/g) and *K*_*L*_ (l/mg) is the constant of Langmuir’s isotherm, which shows the binding affinity between the uranium ions and the tested beads.

The separation factor (*R*_L_) was calculated using Eq. (5):

5 $${R}_{\mathrm{L}}=1/\left(1+{C}_{\mathrm{i}}\times {K}_{\mathrm{L}}\right)$$

where the output value of *R*_L_ could indicate the degree of adsorption possibility between uranium and algal beads as follows:

The adsorption isotherm process is favorable when 0 < *R*_L_ > 1. While it was unfavorable when *R*_L_ < 1, linear *R*_L_ = 1, or irreversible when *R*_L_ = 0 (Malik 2004).

### The Freundlich isotherm

Freundlich isotherm model is a mathematical expression for the adsorption equilibrium between a fluid (liquid or gas) and a solid material assuming the heterogeneity of the surface and interaction between the adsorbed molecules. The Freundlich equation is an empirical expression representing the isothermal variation of adsorption of a liquid or gas onto the surface of solid material, derived by Freundlich (1909) as an empirical relation.

For adsorption of a liquid, the relation between the adsorbed amount per gram of the solid at equilibrium *q*_e_ (mg/g) and the concentration (*C*_e_) in solution at the equilibrium (mg/l) is given by the following equation:

6 $${q}_e={K}_{\mathrm{f}}\times {C_{\mathrm{e}}}^{1/\mathrm{n}}$$

in which *K*_f_ and *n* are constants at a given temperature. When the Freundlich equation is written in logarithmic form, a linear relation between log *q*_e_ and log *C*_e_ is obtained:

7 $$\log\ {q}_e=\log\ {k}_{\mathrm{f}}+\frac{1}{nf}\log\ {\mathrm{c}}_{\mathrm{e}}$$

“*K*_f_”[(mg/g)(l/mg)^1/n^] and *n*_*f*_ are constants related to the adsorption process such as adsorption capacity and intensity, respectively.

Freundlich isotherms are often used to describe adsorption equilibria between a membrane and a feed solution. This is essential for the description of phenomena such as membrane fouling (Van der Bruggen et al. 2002) and breakthrough effects due to desorption (McCallum et al. 2008) in aqueous solutions.

### Adsorption kinetic models

In the present study, pseudo-first-order and pseudo-second-order kinetic models have been attempted to fit the present data. The pseudo-first-order or Lagergren kinetic rate equation is expressed as follows (Kadimpati 2017):

8 $$\frac{\mathrm{dq}}{\mathrm{dt}}=k_1\left(q_{\mathit e}-q_{\mathit t}\right)$$

where “*q*_e_” is the amount of uranium adsorbed at equilibrium per unit mass of adsorbent (mg/g) and “*q*_t_” is the amount of uranium adsorbed at any given time “t” with a constant rate, *K*_l_. The previous equation (Eq. 8) was linearized as follows:

9 $$\ln \left({q}_e-{q}_t\right)=\ln\ {q}_e-{k}_1\ \mathrm{t}$$

The pseudo-second-order reaction model is expressed as follows:

10 $$\frac{t}{q_t} = \frac{1}{k_2{q}_e^2}+\frac{1}{q_e}\mathrm{t}$$

from the linear plots of *t*/*q*_t_ versus *t*, the rate constants *q*_e_ and *k*_2_ and correlation coefficients values were determined.

### Surface characterization and analysis

#### Scanning electron microscopy and energy-dispersive X-ray spectroscopy (SEM-EDX) analyses

The immobilized algal beads were investigated under JSM-T100 scanning microscope (Japan), after fixed on a sample holder with carbon patches, and then covered with carbon layer for 1 min or with a 5–10 μm gold layer using an Edwards Sputter Coater S150B (BOC Edwards, Wilmington, MA, USA (Sarada et al. 2014), together with energy-dispersive X-ray spectroscopy (EDX).

#### Attenuated total reflectance-Fourier-transform infrared spectroscopy (ATR-FTIR)

The ALPHA FTIR spectrophotometer (SN. 100523, Bruker, USA) was used to perform the infrared spectroscopy analysis. For the different algal beads, the spectra were collected in the range of 400 to 4000 cm^−1^ (Belattmania et al. 2020).

### Statistical analysis

The experiments were set as three biological replicates (as mentioned above). The data were represented as mean ± standard deviation (SD). The SPSS software program (version 10, Richmond, Virginia, USA) was used for the comparison of the mean of the data (one-way analysis of variance (ANOVA) with Duncan’s multiple range tests) at *P* < 0.05.

## Results

In this work, different factors including uranium concentration, pH, contact time, and algal biomass dose were applied to the immobilized *Scenedesmus* sp. and *Nostoc* sp. beads to find the optimum condition for uranium biosorption.

### Effect of initial concentration on uranium biosorption

**Fig. 1.**
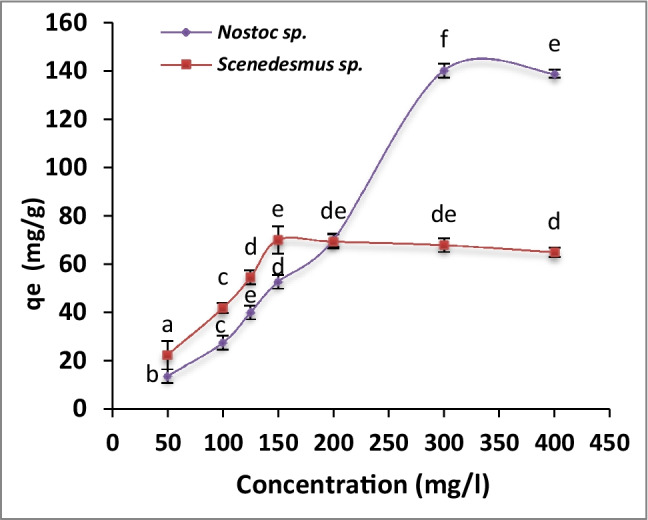
Effect of initial uranium concentration on uranium biosorption capacity of immobilized *Scenedesmus* sp. and *Nostoc* sp. The values represent the mean ± standard deviation (SD) of three replicates. The similar letters represent insignificant differences at *P* < 0.05 (Duncan’s multiple range test)

The data showed that uranium removal by algae was dependent on the initial concentration of uranium till reaching equilibrium (Fig. 1). The maximum removal of uranium (*q*_e_ = 70.07 and 140.14 mg U/g FW) was obtained at 150 and 300 mg/l by *Scenedesmus* sp. and *Nostoc* sp. respectively. After equilibrium, biosorption of uranium was slightly decreased to reach 68.0 and 138.7 mg/g at a concentration of 300 and 400 mg U/l by *Scenedesmus* sp. and *Nostoc* sp. respectively.

### Effect of pH

**Fig. 2.**
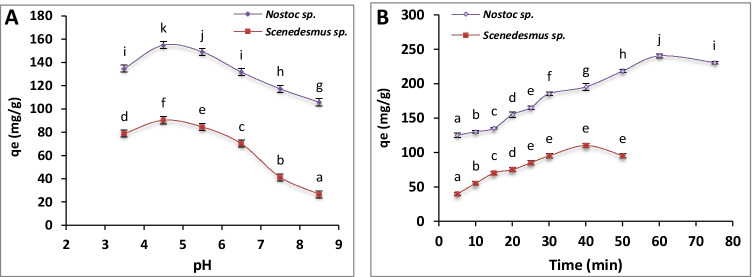
Effect of pH (**A**) and contact time (**B**) on uranium biosorption capacity of immobilized *Scenedesmus* sp. and *Nostoc* sp. The values represent the mean ± SD of three replicates. The similar letters (for each parameter) represent insignificant differences at *P* < 0.05 (Duncan’s multiple range test)

The uranium uptake was varied at a different range of pH (3.5–8.5; Fig. 2A). The optimum pH for uranium removal was 4.5, where the maximum uptake reached 90.3 mg U/g by *Scenedesmus* sp. and 154.6 mg U/g by *Nostoc* sp. The increase of pH resulted in a reduction of uranium uptake by both algae. The lower value for uranium uptake by *Scenedesmus* sp. (26.7 mg/g) and *Nostoc* sp. (105.5 mg/g) was recorded at pH 8.5.

### Effect of contact time

The uranium uptake was increased by increasing contact time till reaching equilibrium (Fig. 2B). The equilibrium was achieved after 40 and 60 min by *Scenedesmus* sp. (110 mg/g) and *Nostoc* sp. (241 mg/g), respectively. The uranium uptake was slightly decreased after reaching equilibrium.

### Effect of algal biomass dosage

**Fig. 3.**
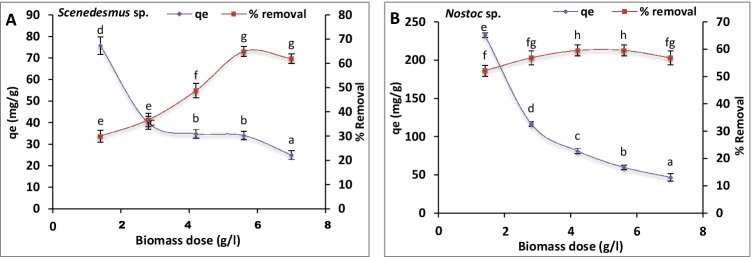
Effect of algal biomass dose on uranium biosorption capacity (mg/g) and uranium removal (%) by *Scenedesmus* sp. (**A**) and *Nostoc* sp. (**B**). The values represent the mean ± SD of three replicates. The similar letters (for each parameter) represent insignificant differences at *P* < 0.05 (Duncan’s multiple range test)

The data revealed that increasing algal biomass favored the uranium removal till reaching equilibrium (Fig. 3). In the case of *Scenedesmus* sp., the uranium uptake (*q*_e_) decreased from 75.8 down to 25 mg U/g FW by increasing the algal biomass from 1.4 to 7 g FW/l, respectively, whereas the maximum removal (65%) of uranium was recorded at 5.6 g FW/l (Fig. 3A).

In the case of *Nostoc* sp., *q*_e_ decreased from 223.5 down to 47 mg U/g FW by increasing the algal biomass from 1.4 to 7 g FW/l, respectively. The maximum removal (60 %) of uranium was recorded at 4.2 and 5.6 g FW/l of *Nostoc* sp. (Fig. 3B).

### Optimization of conditions

The removal of uranium by immobilized *Scenedesmus* sp. and *Nostoc* sp. was reached 65 and 60%, respectively, under the optimized conditions (as recommended by the above experiments).

### Interference by metal ions affecting uranium sorption

#### Effect of sodium sulfate

**Fig. 4.**
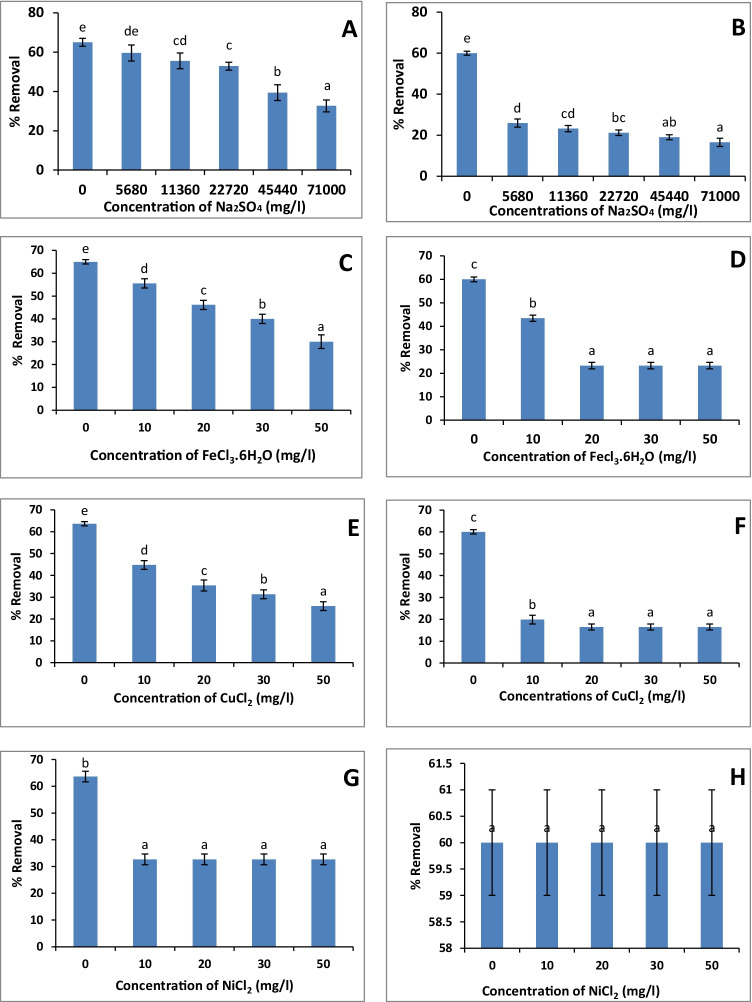

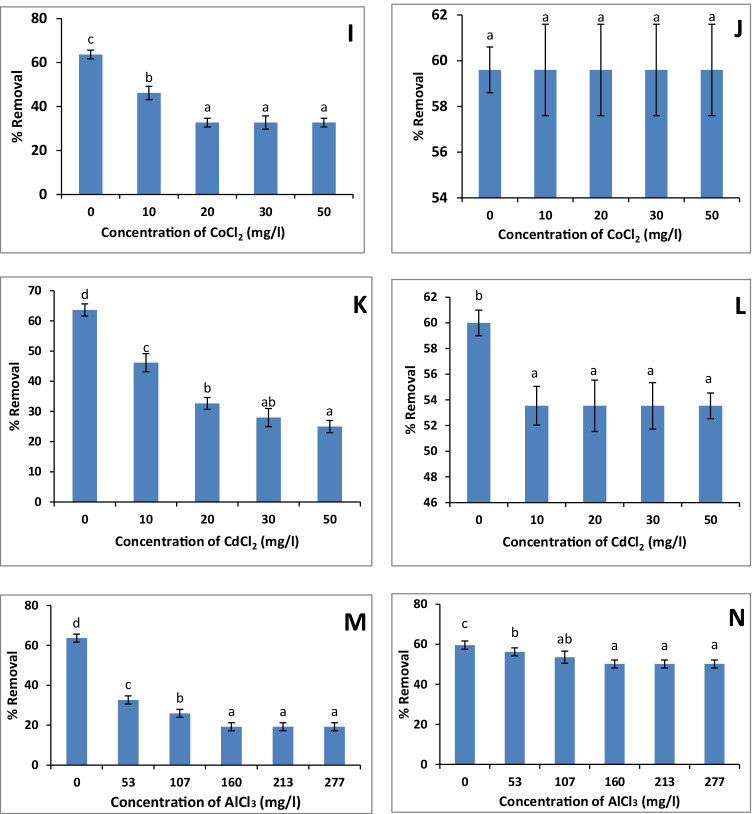
Effect of different concentrations of metal ions (**A**, **B**: Na_2_SO_4_; **C**, **D**: FeCl_3_.6H_2_O; **E**, **F**: CuCl_2_; **G**, **H**: NiCl_2_; **I**, **J**: CoCl_2_; **K**, **L**: CdCl_2_; **M**, **N**: AlCl_3_) on uranium biosorption by *Scenedesmus* sp. and *Nostoc* sp., respectively. The values represent the mean ± SD of three replicates. The similar letters (for each parameter) represent insignificant differences at *P* < 0.05 (Duncan’s multiple range test)

The different concentrations of sodium sulfate had an inhibitory effect on uranium removal by *Scenedesmus* sp. and *Nostoc* sp. (Fig. 4A, B). The lower percent of uranium removal (32.66 and 16.5 %) was recorded at the highest Na_2_SO_4_ concentration (71000 mg/l) by both *Scenedesmus* sp. and *Nostoc* sp. as compared with their control (65 and 60%) respectively.

#### Effect of ferric ions

In the case of *Scenedesmus* sp., a gradual decrease in uranium removal was observed by increasing the Fe^+3^ concentrations (Fig. 4C). The lowest removal value (30%) was recorded at the lowest concentration (50 mg/l Fe^+3^) compared with the control (65%; Fig. 4C). For *Nostoc* sp., the lowest value (23.2 %) was recorded by 20 mg/l Fe^+3^ as compared with control (60%; Fig. 4D). After that, there was no significant (*P* < 0.05) change on uranium removal by *Nostoc* sp. recorded by higher concentrations of Fe^+3^.

#### Effect of copper ions

Similarly, the Cu^2+^ ions had an antagonistic effect on uranium uptake by the tested algae (Fig. 4E, F). In the case of *Scenedesmus* sp., uranium uptake was gradually decreased by increasing Cu^2+^ concentrations till reaching 26% at 50 mg/l Cu^2+^ compared to the control (65%; Fig. 4E), while all the tested Cu^2+^ concentrations (10–50 mg/l) had an inhibitory effect on uranium removal (20%; Fig. 4F) by *Nostoc* sp. compared with control (60%).

#### Effect of nickel ions

The presence of different concentrations of Ni^2+^ (10–50 mg/l) had an antagonistic effect on uranium removal by *Scenedesmus* sp. (32.66 %) compared with control (65%; Fig. 4G). Interestingly, the biosorption of uranium by *Nostoc* sp. was not affected by the presence of Ni^2+^ ions (Fig. 4H).

#### Effect of cobalt ions

The presence of different concentrations of Co^2+^ (10–50 mg/l) had an inhibitory effect on uranium removal by *Scenedesmus* sp., which was almost constant (32.66 %) at the range of 20–50 mg/l Co^2+^ (Fig. 4I). Meanwhile, the biosorption of uranium by *Nostoc* sp. was not influenced by the presence of Co^2+^ ions (Fig. 4J).

#### Effect of cadmium ions

The different concentrations of Cd^2+^ showed a significant antagonistic effect on uranium removal by the tested algae. In the case of *Scenedesmus* sp., the uranium uptake was gradually decreased by increasing Cd^2+^ concentrations till reaching 25% at 50 mg/l Cd^2+^ (Fig. 4K), while, in the case of *Nostoc* sp., all the Cd^2+^ concentrations (10–50 mg/l) had a constant inhibitory effect (53.4%) on the uranium removal (Fig. 4L).

#### Effect of aluminum ions

The antagonistic effect of Al^+3^ on uranium biosorption by the algae was also recorded (Fig. 4M, N). The uranium uptake was gradually decreased by increasing Al^+3^ ions down to 19.2 and 50.1 % (at 160 mg/l Al^+3^) by *Scenedesmus* sp. and *Nostoc* sp., respectively). After that, the uranium uptake by algae was constant.

### Adsorption isotherm models

**Fig. 5.**
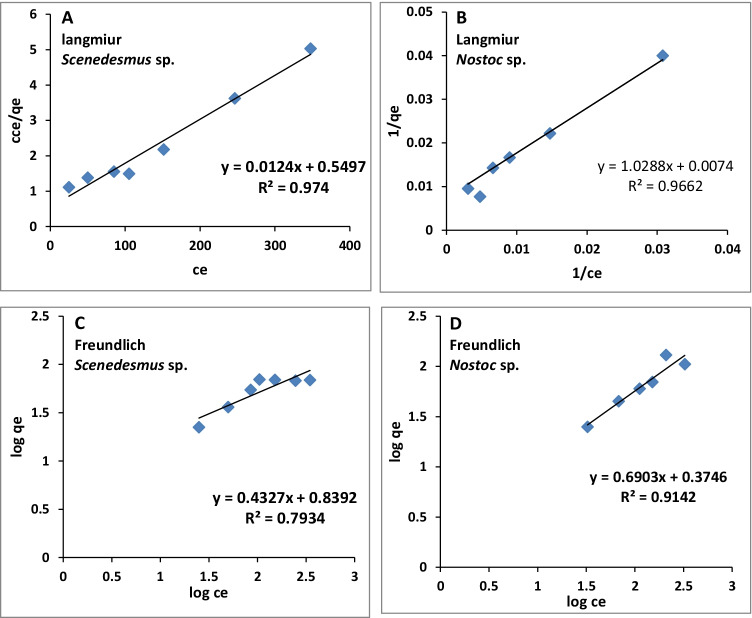
Langmuir (**A** and **B**) and Freundlich (**C** and **D**) isotherm curves for adsorption of uranium by *Scenedesmus* sp. and *Nostoc* sp., respectively

The data obtained from adsorption isotherms is fitted to the linearized form of Langmuir and Freundlich isotherms (Fig. 5, Table 1) as follows:

**Table 1.** The Langmuir and Freundlich isotherm parameters for the biosorption of uranium by *Scenedesmus* sp. and *Nostoc* sp.

Algae	Langmuir				q_max,exp_ (mg/g)	Freundlich		
	*R*^2^	*K*_L_ (l/mg)	*R*_L_	*q*_max,cal_ (mg/g)		*R*^2^	*k*_f_	*n*_f_
*Scenedesmus* sp.	0.98	0.0182	0.268	80	75	0.79	6.9	2.3
*Nostoc* sp.	0.97	0.0072	0.316	135	130	0.91	2.3	1.45

The values of correlation coefficient (*R*^2^), *K*_l_, and *q*_max_ are used to describe the adsorption process and the applicability of the equation of isotherm (Table 1). The data is adapted to the linearized form of the Langmuir model. The experimental *q*_max_ (*q*_max,exp_) of *Scenedesmus* sp. was 75 (mg/g) and the calculated value (*q*_max,cal_) was 80 (mg/g); *R*^2^ reached 0.98; the Langmuir constant (*K*_L_) and the separation factor (*R*_L_) parameters were 0.0182 and 0.268. In case of *Nostoc* sp., the *q*_max,exp_ and *q*_max,cal_ were coordinated (130 and 135 mg/g, respectively), while *R*^2^ reached 0.97 and *K*_L_ and *R*_L_ were 0.0072 and 0.316. Regarding to the Freundlich model, *R*^2^, *K*_f_, and *n*_f_ for *Scenedesmus* were 0.79, 6.9, and 2.3, respectively. Meanwhile, *R*^2^, *K*_f_, and *n*_f_ were 0.91, 2.3, and 1.45 for *Nostoc* sp., respectively (Table 1).

### Adsorption kinetic models

**Fig. 6.**
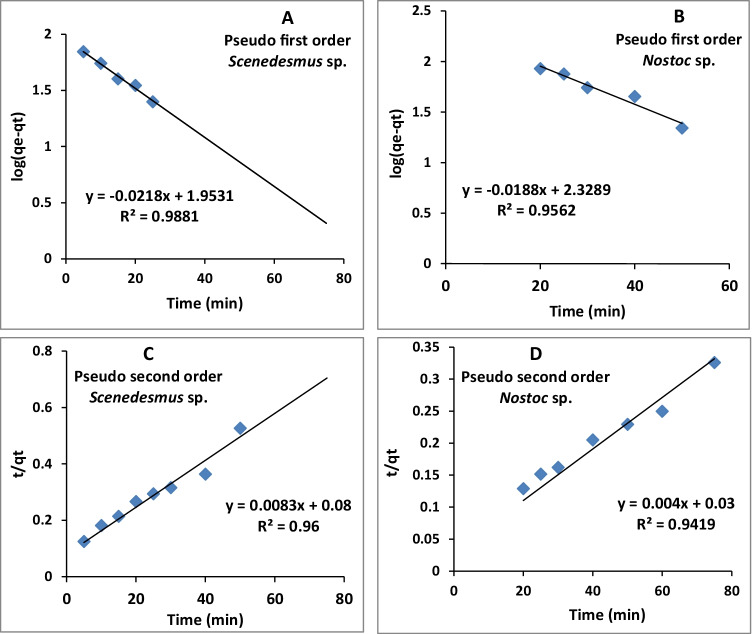
Pseudo-first (**A** and **B**) and pseudo-second (**C** and **D**) order kinetics of uranium sorption by *Scenedesmus* sp. and *Nostoc* sp., respectively

In the present study, the present data were attempted to fit into the pseudo-first-order and pseudo-second-order kinetic models (Fig. 6, Table 2). From the linear plot between log (*q*_e_-*q*_t_) and *t* (min), the calculated *q*_max_ (*q*_max,cal_) of *Scenedesmus* sp. and *Nostoc* sp. were 89 and 213 mg/g, whereas the experimental values (*q*_max,exp_) were 110 and 241.24 mg/g, respectively. *K*_L_ and *R*^2^ were 0.05 and 0.98 for *Scenedesmus* sp. and 0.0423 and 0.956 for *Nostoc* sp*.*, whereas the parameters of the pseudo-second-order model, i.e., *K*_2_, *R*^2^, and *q*_max_, can be calculated from plotting linear relation between *t*/*q*_t_ and *t*. The *q*_max,cal_ was more close to the value of *q*_max,exp_ for the tested algae. The *q*_max,cal_ of *Scenedesmus* sp. and *Nostoc* sp. were 120 and 250 mg/g, whereas the *q*_max,exp_ values were 110 and 241.2 mg/g, respectively. The correlation coefficient *R*^2^ and *K*_2_ constant were 0.97 and 8.68×10^−4^ for *Scenedesmus* sp. and 0.96 and 5.33×10^−4^ for *Nostoc* sp. (Table 2). The calculated data for pseudo-second-order were more close to the experimental one and so the applicability of the pseudo-second-order model was valid for both tested algae.

**Table 2.** Kinetic parameters for the biosorption of uranium by the *Scenedesmus* sp. and *Nostoc* sp.

Algae	Pseudo-first-order			*q*_max,exp_ (mg/g)	Pseudo-second-order		
	*K*_1_	*R*^2^	*q*_max,cal_ (mg/g)		*K*_2_	*R*^2^	*q*_max,cal_ (mg/g)
*Scenedesmus* sp.	0.05	0.98	89	110	8.68×10^-4^	0.97	120
*Nostoc* sp.	0.0423	0.956	213	241.24	5.33×10^-4^	0.96	250

### Biomass characterization

#### ATR-FTIR analysis

The ATR-FTIR spectra of untreated algal beads were compared with the spectra of beads after uranium biosorption to detect the observable differences and define the functional groups that participated in uranium biosorption.

**Fig. 7.**
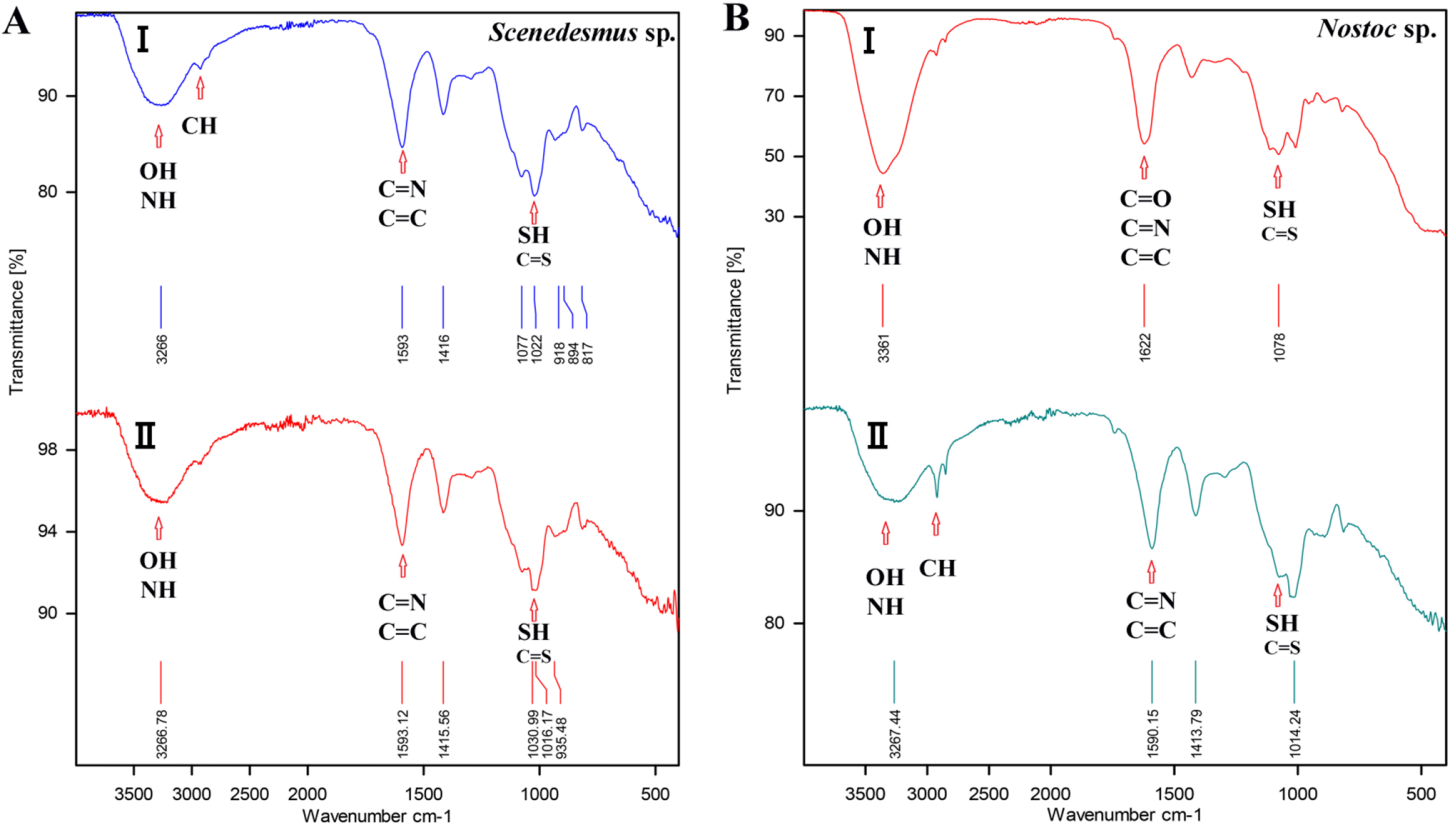
ATR-FTIR spectra of immobilized (**A**, *Scenedesmus* sp., and **B**, *Nostoc* sp.) algae before (I) and after (II) uranium treatment, under the optimized conditions

In the spectra of untreated *Scenedesmus* beads, the peaks appeared at 3266 cm^−1^ representing OH and NH; 2926 cm^−1^ representing CH aliphatic; 1593 cm^−1^ representing CN and CC; and 1022 cm^−1^ representing CS and SH. The immobilized treated *Scenedesmus* alga showed intensive peaks at 3266.78 cm^−1^ representing OH and NH; 1593.12 cm^−1^ representing CC and CN; and 1030–1016 cm^−1^ representing CS and SH (Fig. 7A).

In the case of untreated immobilized *Nostoc* alga, it showed intense peaks at 3361 cm^−1^ representing OH and NH; 1622 cm ^−1^ representing CO, CN, and CC; and 1078 cm^−1^ representing SH and CS, while the peaks of treated immobilized *Nostoc* appeared at 3267 cm^−1^ representing OH and NH; 2921 cm^−1^ representing CH aliphatic; 1590 cm^−1^ representing CN and CC; and 1014 cm^−1^ representing CS and SH (Fig. 7B).

#### SEM-EDX analyses

**Fig. 8.**
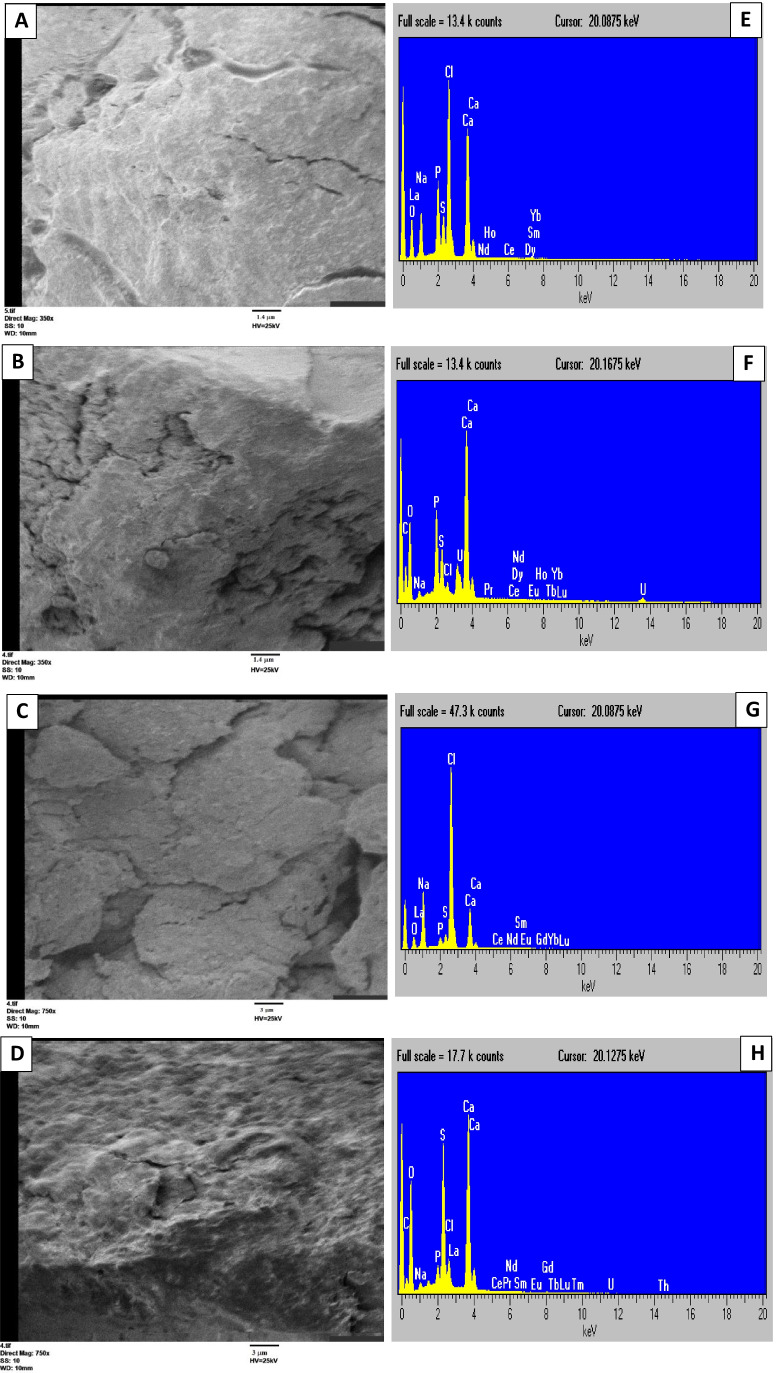
The left panel; SEM images of *Nostoc* sp. (before, **A** and after, **B**; Mag-350X) and *Scenedesmus* sp. (before, **C** and after, **D**; Mag-750X) beads treated with uranium. The right panel; EDX images of *Nostoc* sp. (before, **E** and after, **F**) and *Scenedesmus* sp. (before, **G** and after, **H**) beads treated with uranium

The images of scanning electron microscopy (SEM) of the immobilized *Nostoc* and *Scenedesmus* algae treated with uranium were relatively rough, irregular, and heterogeneous with obvious cracks and pores (Fig. 8A, C), while the untreated beads had smooth and more uniform surfaces (Fig. 8B, D).

The energy-dispersive X-ray (EDX) is a technique for detecting the presence of elements or metallic ions that present in the specimen (algal biomass) or absorbed on its surface. The EDX spectra of treated immobilized *Nostoc* and *Scenedesmus* cells displayed a clear identifiable uranium peak (Fig. 8F, H), which was absent in the untreated samples (Fig. 8E, G). This confirmed the occurrence of uranium biosorption and accumulation on the surface of algal beads as compared with control. Additionally, other peaks of Ca, Na, O, S, P, and C were also observed on the surface of algal biomass of both algae (before and after U treatment).

## Discussion

### Factors affecting biosorption of uranium by immobilized algae

#### Effect of initial uranium concentration

The removal of uranium was largely dependent on initial metal concentration till the equilibrium (Fig. 1). After equilibrium, biosorption of uranium was slightly decreased as a result of saturation. This was in agreement with Bayramoglu et al. (2015) who reported the increase of adsorption rate by increasing the initial concentration of uranium up to the saturation point. In this context, Amini et al. (2013) reported the reduction of uranium removal (from 97.65 to 89.69 %) by increased uranyl ions in the aqueous solution (from 100 to 300 mg/l) by *C. vulgaris*. Also, Mehta et al. (2002) stated that removal of heavy metal was increased by increasing of initial metal concentration till reaching equilibrium. A possible explanation for this phenomenon is that the initial concentration of metal ions improves the driving force to overcome the resistance of mass transfer between biosorbent and fluid (liquid) phase’s bulk. Additionally, initial concentration improves the biosorption process by increasing collisions between biosorbent and metal ions (Bayramoglu et al. 2015). After the saturation point (equilibrium), the competition between the uranyl ions on binding sites of the biosorbent led to a reduction in the rate of uranium biosorption (Kolhe et al. 2020).

In this study, the superiority of immobilized *Nostoc* against *Scenedesmus* beads may be related to the high affinity of cyanobacteria to adsorb uranyl ions, to the adaptability to sequester uranium from its aqueous solution, or to the significant biochemical composition for the favor of metal-adsorption process (Cecal et al. 2012; Vijayaraghavan et al. 2018; Yuan et al. 2020).

#### Effect of pH

The pH value is one of the most primary factors affecting on biosorption process by algae, as a result of its direct effect on solubility and toxicity of heavy metals in wastewater (Bayramoglu et al. 2015). Brinza et al. (2007) showed the effect of pH on metal speciation and algal tolerance, especially the pH effect on metal-binding sites on the cell surface, and metal chemistry in water. They reported the favorable pH range for the biosorption of most heavy metals to be 3–6.5 by the dead biomass of macroalgae. They argued that, at this range, the chemistry of heavy metals was suitable as they are in high soluble ion form. The present data showed that optimum pH for maximum uranium removal was 4.5 (Fig. 2A). For uranium, in particular, this was also supported by the literature, where the optimal pH for uranium adsorption on algal biomass was recorded between 4.0 and 5.0 (Bayramoglu et al. 2015). Erkaya et al. (2014) reported that the biosorption of uranyl ions by free *C*. *reinhardtii* cells, entrapped algal cells, and bare CMC beads were highly maximum at pH 4.5 which decreased below or above that point.

The behavior of functional groups on the algal cell surface and the complex formation with metal ions are largely influenced by pH. At pH lower than 3, the competition between hydrogen and uranyl ions was intense for the binding sites, which led to a reduction of metal biosorption by the algal beads (Brinza et al. 2007; Yu et al. 2014). Moreover, the availability of metal-binding groups was also affected because most of these groups are acidic (e.g., carboxyl group) and present in the protonated state at acidic pH, where repulsive forces exist between them and heavy metal cations, therefore, decrease the biosorption capacity (Bilal et al. 2018).

On the other hand, the increase of pH resulted also in a reduction of uranium uptake by both algae. Several factors may participate in this result, for example, the heavy metals generally tend to precipitate in hydroxides form at higher pH (≥ 6.5) left small quantity to be adsorbed by the algal biomass (Brinza et al. 2007; Bilal et al. 2018). Also, the disruption between ligands containing phosphate, carboxyl, imine, and amino groups on the surface of the algal beads may occur at high pH, as these legends usually have pKa values in the range of 4.0–7.0 (Yu et al. 2014; Bayramoglu et al. 2015).

#### Effect of contact time

The biosorption rate of uranium by the immobilized algae was monitored through the reduction of metal concentration with time. Initially, the biosorption rate of uranium was high and the saturation levels were achieved after 40 and 80 min, for *Scenedesmus* and *Nostoc*, respectively (Fig. 2B). After the saturation, the biosorption rate of uranium started to slightly decrease. The initial increase of biosorption may be related to the availability of binding sites on the surface of the algal biomass, which reduced by time, as these sites saturated with uranium (Bhat et al. 2008). This was agreed with previous reports despite the differences in the recorded values. For example, the uptake of UO_2_^2+^ by *B*. *braunii* was increased by time, where the optimum U uptake was recorded at 74 min (Celik et al. 2019). Similarly, the biosorption of uranyl ions by free-living biomass of *N*. *linckia*, *S. platensis*, and *Porphyridium cruentum* was time-dependent up to consistency at 24–48 h (Cecal et al. 2012).

As to the combined forms of algae with chemical matrix, Bayramoglu et al. (2015) reported that the PEI and amidoxime-modified *S. platensis* biomasses adsorb uranyl ions by a rate of 70 % after 40 min, while the equilibrium was reached after 60 min (Bayramoglu et al. 2015). Erkaya et al. (2014) found that the free, entrapped *C. reinhardtii* cells and bare CMC beads showed an initial increment of the biosorption uranyl ions up to 30 min; following that, the biosorption process reached the equilibrium in 60 min. Nonetheless, the time to attain the equilibrium was notably proportional to the initial concentration of the uranyl ions (Jiang et al. 2020).

Therefore, the sorption of uranyl ions exists in two stages, a first rapid one (surface adsorption) followed by a slow intracellular diffusion (Bhat et al. 2008; Erkaya et al. 2014). On this basis, the different values of the optimum time for each alga (Fig. 2B) may be explained by the difference between their biosorption rate, where the rapid surface adsorption and the intracellular diffusion were different due to the disparity of their cell wall, cellular, and biochemical compositions, etc. For example, the cyanophyte *Anabaena flos-aquae* was reported to biosorb uranyl ions at the first 20 min, and then reached the equilibrium (*q*_e_ 196.4 mg/g) after 50 min, whereas the maximum biosorption (95.6 %) of uranyl ions by the green alga *Parachlorella* sp. was obtained within 60 h. It follows a rapid rate (from 0.5 to 4 h) followed by a slight increase one (4–24 h); thereafter, the biosorption rate was then stabilized (Yoon et al. 2021).

For other heavy metals, Ahmad et al. (2018) reported the saturation time for the biosorption rate of Fe^2+^, Mn^2+^, and Zn^2+^ ions by free **(**240 min) and immobilized (300 min) *C. vulgaris* biomass. Furthermore, they found that immobilized *C. vulgaris* biomass exceeded the free biomass algal-form in terms of biosorption rates of the tested metal ions.

#### Effect of algal biomass dosage

The biosorption of uranium (mg U/g FW) was found to increase by increasing of biosorbent dose (algal biomass) up to a constant level at high dosages (Fig. 3). This increment may be due to an increase of surface area and excess of available binding sites provided by the higher biosorbent dose (Erkaya et al. 2014; Yu et al. 2014; Bayramoglu et al. 2015; Ahmad et al. 2018). Moreover, the results (Fig. 3) showed also that removal of uranium was inversely proportional to the algal dose. In this regard also, Smječanin et al. (2022) reported the reduction in the adsorption capacity by the increase of the biocomposite mass. This could be due to the formation of biomass aggregates (at high doses) that affect the active surface area of the biosorbent and may led to reduced active binding sites of the applied biosorbent (Sarı and Tuzen 2008; Smječanin et al. 2022).

Both metal uptake capacity and biosorption efficiency are important equally because they are used in describing the sorption performance of the investigated biosorbent (Vijayaraghavan et al. 2007). The relationship between biomass dose and sorption was affected by availability of metal-binding sites, binding site interference, reduction of uniformity at high biomass doses, and electrostatic interactions between groups (Mehta and Gaur 2005). For example, increasing the biosorbent concentration up to 40 g/l resulted in decreasing in copper removal (Bishnoi and Pant 2004). Also, an obvious reduction in the removal of lead was reported by increasing the biomass of *Spirulina maxima* from 0.1 to 20 g/l (Gong et al. 2005). Yet, the maximum biosorption efficiency occurs at a lower biomass dosage of biosorbents.

### Interference of metal ions on uranium biosorption

It is important to note that the presence of other adsorbable ions in uranium solution may affect the biosorption process by competing on active binding sites on the cell surface, reducing the binding of other ions, or preventing uranium removal (Amini et al. 2013).

The results of the present study showed an inhibitory effect of Na_2_SO_4_, Fe^3+^, Cu^2+^, Ni^2+^, Co^2+^, Cd^2+^, and Al^3+^ on uranium biosorption by *Scenedesmus* sp., while the biosorption of *Nostoc* was inhibited only by Na_2_SO_4_, Fe^3+^, Cu^2+^, Cd^2+^, and Al^+3^, while Ni^2+^ and Co^2+^ had no significant effect (Fig. 4). Similar results were obtained by Hu et al. (1996) who found an inhibitory effect of metal ions on uranium binding. The metals are arranged as (in order of inhibition) Fe^3+^ > A1^3+^ > Pb^2+^ > Cu^2+^ > Cr^3+^ > Cd^2+^, Mn^2+^, Ba^2+^, Co^2+^. They also reported that SO_4_^−2^ in addition to Na^+^, Cl^−^, NO_3_^−^, and acetate had a negligible effect on uranium biosorption, which was inconsistent with our results (Fig. 4). Amini et al. (2013) also confirmed the inhibitory effect of aluminum on uranium sorption. Similarly, the biosorption of lead was inhibited by Cu^2+^, while Zn^2+^ had a negligible effect (El-Naas et al. 2007).

Likewise, the removal percentage of metal of interest, which is uranium ions in this case, was decreased by the increment of other metal ions as a result of their interaction (Han et al. 2008). In this regard, Zhang et al. (1997) reported that Cu^2+^, Ni^2+^, Zn^2+^, Cd^2+^, and Mn^2+^ competed slightly with uranyl removal using *Scenedesmus obliquus*. Therefore, the competition of ions for binding sites of the biosorbent, complexation, and/or their antagonistic effect led to an inhibitory effect on uranium biosorption (Zhang et al. 1997; El-Naas et al. 2007).

### Adsorption isotherms

The way that adsorbates interact with adsorbents at constant pH and temperature is described by the adsorption isotherm. The investigation of metal uptake by isotherm models is an essential study that provides information about adsorbent capacities, adsorption process, characters of adsorbent surface, design of more efficient and successful treating system, and the explanation to optimize adsorption process mechanism (Sahoo and Prelot 2020). Two applicable models were commonly used: the Langmuir and Freundlich models. The Langmuir isotherm assumes that adsorbate and adsorbent are in dynamic equilibrium in monolayer adsorption. The model assumptions include (1) homogeneity of the surface, (2) monolayer adsorption of adsorbates on the surface, (3) no interaction between adsorbed molecules, and (4) a reversible nature of the adsorption process. That is, it considers the sorbate is bound uniformly and consistently on the sorbent’s surface, i.e., dynamic equilibrium (El-Naas et al. 2007; Yuan et al. 2020), whereas the Freundlich isotherm is used to explain the adsorption at a heterogeneous surface. It assumes that adsorption occurred in a multilayered manner, non-ideal, reversible, with different energies of the binding sites (Sahoo and Prelot 2020).

The results in Table 1 showed that the Langmuir model was more adapted to describe the biosorption of uranium by immobilized *Scenedesmus* and *Nostoc* algal cells, compared with the Freundlich model. This may be attributed to the higher value of equilibrium parameters, and the values of *R*_l_ were between 0 and 1 (Malik 2004). Moreover, the values of *q*_max,exp_ and *q*_max,cal_ in the Langmuir model were much closer, as compared to that of Freundlich isotherm. Also, the *n*_f_ value in Freundlich isotherm was below 1.0 for both tested algae and therefore it is considered less favorable than the Langmuir model (Malik 2004; Kadimpati 2017). The values of *q*_max,cal_ were 80 and 135 mg/g for *Scenedesmus* sp. and *Nostoc* sp., respectively (Table 1), which are acceptable rates for uranium biosorption (Amini et al. 2013). Previously, the biosorption of uranyl ions by *C*. *vulgaris* and *B*. *braunii* was well adapted to the Langmuir model, with *q*_max,cal_ of 165.09 and 67.8 mg/g mg/g, respectively (Amini et al. 2013). Also, the biosorption of uranium by *Anabaena flos*-*aquae* was well fit to the Langmuir model with *q*_max,cal_ of 190.1 mg/g (Yuan et al. 2020).

The applicability of Langmuir isotherm to describe the monolayer adsorption process of uranyl ions by *N*. *linckia*, *S. platensis*, and *Porphyridium cruentum* (Cecal et al. 2012), chitosan-immobilized *C*. *pyrenoidosa* (Jiang et al. 2020; Liu et al. 2022), and immobilized marine yeast *Yarrowia lipolytica* (Kolhe et al. 2020) was also verified.

In this regard also, Erkaya et al. (2014) described uranium absorption by free, entrapped *C*. *reinhardtii* cells, and bare CMC beads to follow the Langmuir isotherm model, and the values of *q*_max,cal_ were 344.4, 232.6, and 192.3 mg/g, respectively. However, Zhang et al. (1997) reported that removal of uranyl ions by *Scenedesmus obliquus* followed the Freundlich adsorption isotherm and the maximal binding capacity was 75 ± 5 mg/g dry weight (DW) at 28 ± 3 °C.

In respect to other metals, the biosorption of Fe^2+^, Mn^2+^, and Zn^2+^ was well fitted to the Langmuir model by both free (*q*_max,cal_ of 78.64, 72.71, and 70.26 mg/g) and immobilized (133.06, 121.81, and 114.57 mg/g) forms of *C. vulgaris* biomass, respectively (Ahmad et al. 2018).

### Adsorption kinetics models

The time required to attain equilibrium between adsorbates and adsorbent is determined using kinetic models that provide information about the pathway of adsorption and propose mechanisms regarding the biosorption process (Sahoo and Prelot 2020). Two fundamental models were used for the previous aim; firstly, the pseudo-first-order kinetic model given by Lagergren (Brinza et al. 2007; Kadimpati 2017) considers that there is a direct relationship between the rate changes of solute uptake with time and saturation concentration changes and solid uptake amount with time. In other words, it assumes that the number of vacant adsorption sites is proportional to the rate of occupation of those sites (Brinza et al. 2007; Erkaya et al. 2014). When the adsorption process occurs through the diffusion interface, a pseudo-first-order equation is followed. Secondly, the pseudo-second-order kinetic model, where the adsorption capacity is the major factor affecting the adsorption rate, describes the displacement of alkaline-earth ions by metal ions from algal biosorption sites, i.e., describes the electron interactions between molecules of biosorbent and sorbate. Adsorption by this model is assumed to be chemisorption and the behavior of adsorption is predicted. It is characterized by easy calculation of adsorption equilibrium capacity compared with the pseudo-first-order kinetic model (Brinza et al. 2007; Erkaya et al. 2014; Ahmad et al. 2018).

To analyze the kinetics of uranium biosorption by the algal beads, the linear forms of pseudo-first-order and pseudo-second-order models were used to fit the experimental data. The data of the kinetic models in Table 2 showed that the process of uranium biosorption was well adopted by the pseudo-second-order model because of the similar values of both the calculated and experimental biosorption capacity. Moreover, the values of *R*^2^ were of confidence level for both models. Therefore, the uranium biosorption by the investigated algae is assumed to be a rate-limiting process.

Correspondingly, the pseudo-second-order model was valid to uranium biosorption by free *C. vulgaris* (Amini et al. 2013), *C*. *reinhardtii* (free and immobilized; Erkaya et al. 2014), and chitosan-immobilized *C*. *pyrenoidosa* (Jiang et al. 2020; Liu et al. 2022). Moreover, the uranium biosorption by *Anabaena flos-aquae* has also followed the same model, where the calculated (*q*_max,cal_ = 197.71 mg/g) and the experimental (*q*_max,cal_ = 196.4 mg/g) *q*_e_ values were matched, implying the chemisorption mechanism of the adsorption process (Yuan et al. 2020). Likewise, the data of uranyl ion adsorption by native, PEI, and amidoxime-modified *S. platensis* biomasses were well followed by the pseudo-second-order model, where the values of *q*_max,cal_ and *q*_max,exp_ were agreed (Bayramoglu et al. 2015).

For other metals, the bioso rption of pb^2+^ ions by non-living *C. vulgaris* followed also the pseudo-second-order model, where the calculated and experimental values of *q*_e_ **(**45.7 and 45.6 mg/g) were almost typical. Similarly, the biosorption rate of Fe^2+^, Mn^2+^, and Zn^2+^ ions by both the free and Ca-alginate-immobilized *C. vulgaris* biomass followed the pseudo-second-order model (Ahmad et al. 2018).

### Biomass characterization

#### ATR-FTIR

The functional groups responsible for uranium biosorption of the algal beads were OH, NH, CO, CH, CC, CN, CS, and SH, as shown by ATR-FTIR analysis (Fig. 7). Stretches of these alcohols, phenols, carboxylic, amides, thiol, sulfhydryl, or sulfanyl groups, have been shown to aid in the adsorption of uranyl ions to algal cells (Belattmania et al*.* 2020).

It is worth noting that all of the spectral patterns of the different functional groups serve as a distinctive identity of algal cells, and changes after uranium biosorption could be regarded as complexation/interaction of these groups with it during the biosorption process (Vogel et al. 2010; Yuan et al. 2020). For example, the shift of 1735 and 1737 cm^−1^ bands (represent CO groups) to 1744 cm^−1^, after uranium biosorption by *C. vulgaris*, suggests the lipid involvement in uranium interaction. Also, the changes in the wavelength of COOH groups (at 1464 cm^−1^) suggest their implication in the uranium biosorption (Vogel et al. 2010). Likewise, Amini et al. (2013) observed an increase of OH groups’ peak on *C. vulgaris* biomass after uranium biosorption, which may be due to the hydroxylation of some polysaccharides into shorter saccharides. Moreover, the decrease of 1400 cm^−1^ peak (COOH group) and alteration of 1076 cm^−1^ peak (CO group) ensure their participation in uranium adsorption.

Celik et al. (2019) showed that the outer wall of *B*. *braunii* contains high (15–75% DW) hydrocarbon contents, where CH_2_ chains (at 2922 and 2850 cm^−1^) and OH groups (in the range of 3700–3100 cm^−1^) peaks were noticed. These may facilitate uranium biosorption due to their intermolecular bonding. They also recorded the role of carboxylate (observed at 167–1419 cm^−1^) and amino (observed around 1470 cm^−1^) groups for the uranium biosorption process. Additionally, Jiang et al. (2020) confirmed the involvement of NH and OH (at 3431 cm^−1^), stretching vibrations of CO (1655 cm^−1^), CH_2_ stretching, and CH or CH_2_ bending (at 2924 and 1384 cm^−1^) in uranium adsorption by *C. pyrenoidosa*.

Similar to our findings, the significant role of OH, CN, CNC, CO, COO, CONH, NH_2_, SH, CH, CC, CS, and C groups during uranium biosorption was also confirmed for *C. reinhardtii* (Erkaya et al. 2014), *C*. *vulgaris* (Ahmad et al. 2018), and PVA-alginate-*J*. *rubens* matrix (Kadimpati 2017).

#### SEM-EDX

The SEM and EDX analyses are useful tools for the characterization of biosorbents. The changes that appeared on the surface of algal beads of treated samples may be due to the accommodation resulting from the biosorption of uranium, and the potential linkage with varied functional groups (Ghoneim et al. 2014). Saravanan et al. (2011) explained changed surface morphology after metal biosorption to the replacement of metal ions with other surface cations and the formation of strong cross-linkage (ion-exchange mechanism), whereas the cracks on the matrix surface may support uranium adsorption by providing more functional groups per surface area. In addition, the pores on the surface may promote the sorption opportunity of metal ions (Kadimpati 2017; Ahmad et al. 2018).

Furthermore, the appearance of uranium peaks in EDX spectra for the treated algal samples (Fig. 8F, H) is another clue to the biosorption process. It confirms the ability of algal biomass for uranium sequestration (Acharya et al. 2009; Vijayaraghavan et al. 2018), whereas the low peaks of uranium could be due to the low concentration used (Acharya et al. 2009).

The presence of Ca, C, and O peaks (Fig. 8E–H) could be attributed to the varied composition of the algal beads. For example, alginate (alginic acid) is a heteropolysaccharide molecule rich in carbon and oxygen (Pawar and Edgar 2012). Other elements are components of algal cell walls which possess different functional groups (Ahmad et al. 2018). Moreover, the application of CaCl_2_ as a hardening agent during the preparation of algal beads explains the high amplitude of Ca peak in EDX spectra.

## Conclusions

The present work reported the biosorption equilibrium and kinetics of uranium from aqueous solution using immobilized *Scenedesmus* and *Nostoc* sp. algae. Factors like metal concentration, contact time, pH, and biosorbent dosage were significantly affecting the biosorption process. The data showed the superiority of *Scenedesmus* over *Nostoc* sp. for uranium removal (65 and 60%) under the optimum conditions. However, the interaction of metal ions as Na_2_SO_4_, Fe^3+^, Cu^2+^, Cd^2+^, Ni^2+^, Co^2+^, and Al^3+^ did not support the uranium biosorption. The biosorption process was well described by Langmuir isotherm and pseudo-second-order kinetic models. The ATR-FTIR analysis showed the functional groups of the algal beads responsible for uranium biosorption, i.e., OH, NH, CH, CO, CN, CC, SH, and CS groups. Moreover, the SEM and EDX analyses showed the differences of algal beads surface before and after U treatment which may be attributed to the accommodation resulting from the biosorption process. The results confirmed the validity of the tested algae for the biosorption of metal ions and suggest their application on large-scale cost-effective mode for the treatment of aqueous solutions and wastewater.

## Supplementary information

ESM 1 (PDF 927 kb)
