# Supplementary material for: Biosorption of uranium by immobilized Nostoc sp. and Scenedesmus sp.: kinetic and equilibrium modeling
Source: Environ Sci Pollut Res Int. 2022 Jun 30;29(55):83860–77. doi: 10.1007/s11356-022-21641-9 (PMC9643271; doi:10.1007/s11356-022-21641-9)
Supplement: Supplementary file 1 — (PDF 927 kb) [file 11356_2022_21641_MOESM1_ESM.pdf]

Supplementary materials

Mostafa M. S. Ismaiel[1*], Yassin M. El-Ayouty[1], Saad A. Abdelaal[2], Hoda A. Fathey[1]

[1] Department of Botany and Microbiology, Faculty of Science, Zagazig University, Zagazig 44519, Egypt.

[2] Nuclear Research Center, Egyptian Atomic Energy Authority, P.O. Box, 13759, Cairo, Egypt.

* Corresponding author; e-mail: mostafamsami@yahoo.com; permanent address: Department of Botany and Microbiology, Faculty of Science, Zagazig University, Zagazig, 44519, Egypt.

Tel: +2 0111 7373167

Fax: +2 055 2308213

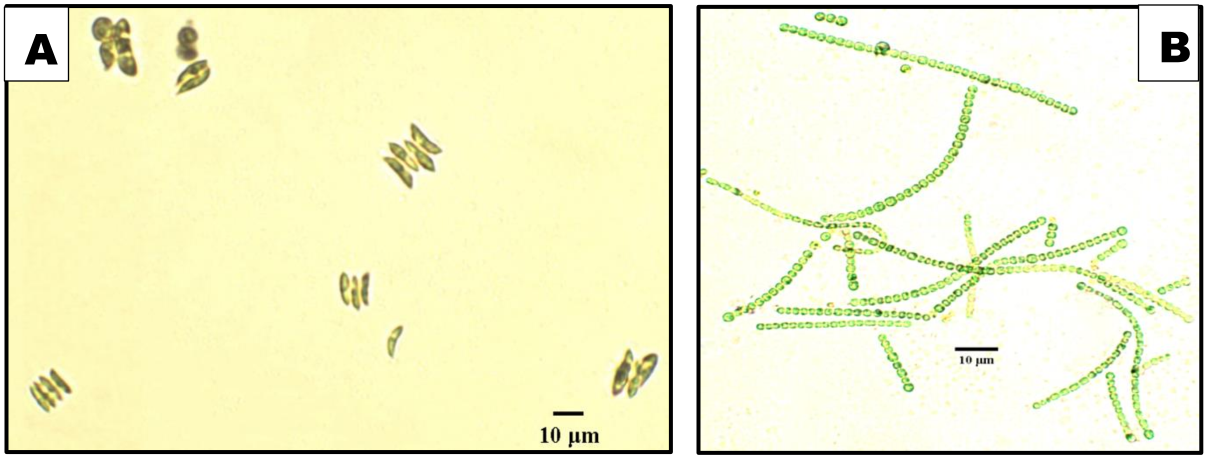

Fig. S1. Microscopic images of the free-form of algae investigated for their biosorption efficiency of uranium (in immobilized form). A, *Scenedesmus* sp.; B, *Nostoc* sp. The images captured with iScope microscope (Euromex iScope series, Holland) with digital camera (CAMERA CMEX 5).

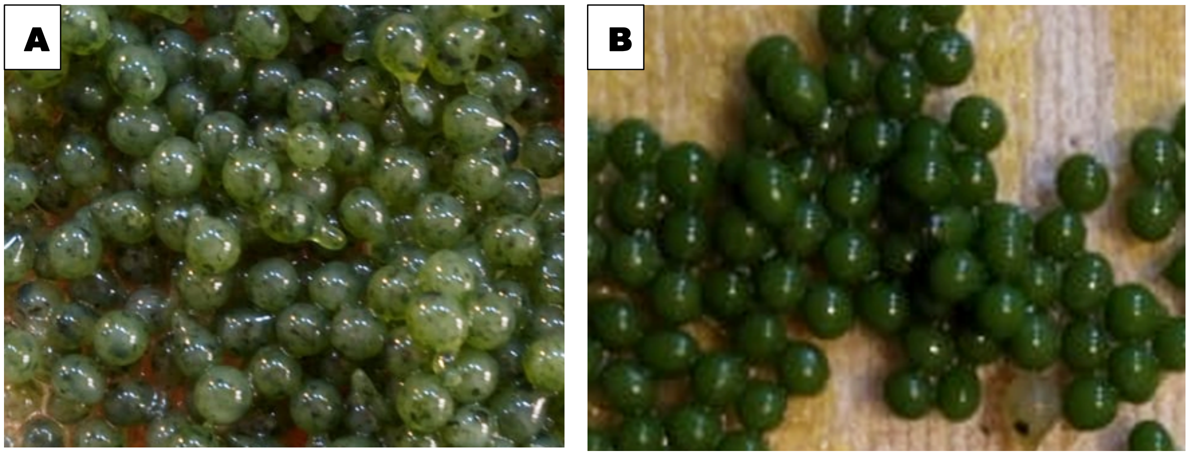

Fig. S2. Immobilized algal cells; A, *Nostoc* sp.; B, *Scenedesmus* sp.

Table S1. Composition of algal culture media

Modified Watanabe medium (El-Nawawy et al., 1958) ($g.l^{-1}$)

0.3 g K_2HPO_4, 0.20g $MgSO_4.7H_2O$, 0.20g K_2SO_4 , 0.1g $CaCO_3$, 2.0g glucose, 0.20 ml of 1% $FeCl_3$ solution (freshly prepared), and 1.0 ml of micronutrient solution.

The micronutrient solution was prepared ($g.l^{-1}$) as:

2.80 g H_3BO4; 0.22g $ZnSO_4.7H_2O$; 0.08g $CuSO_4.5H_2O$; 1.80g $MnCl_2$ and 0.02g $H_2MoO_3.H_2O$, and made up to 1000 ml by distilled water. The pH of the medium was adjusted to 7.4.

BG11 medium (Stanier et al., 1971) ($g.l^{-1}$)

1.5g $NaNO_3$, 0.4g $K_2HPO_{4,}$ 0.75g $MgSO_4.7H_2O$, 0.36g $CaCl_2.2H_2O$, 0.06g citric acid, 0.06g ferric ammonium citrate, 0.01g EDTA-Na_2, 0.2g $Na_2CO_{3,}$ and 1ml of trace metal solution.

The trace metal solution was prepared ($g.l^{-1}$) as:

2.86g H_3BO_4, 1.81g $MnCl_2.4H_2O$, 0.22g $ZnSO_4.7H_2O$, 0.39g $NaMoO_4.5H_2O$, 0.08g $CuSO_4.5H_2O$, and 0.05g $Co(NO_3)_2.6H_2O$. The pH of the medium was adjusted to 7.1.
